# Defining the Patient Journey and Identifying Digital Health Solutions in Treatment-Resistant Schizophrenia

**DOI:** 10.62641/aep.v53i6.1959

**Published:** 2025-12-17

**Authors:** Eva Grasa, Anna Forment, Anna Alonso-Solís, Liliana Ramalho, Alexandra Roldán, Esther Pousa, Susanna Aussó, Jesus Berdun, Ana Genova Tesoro, Joan Escudero, Iluminada Corripio

**Affiliations:** ^1^Mental Health, Sant Pau Research Institute (IR SANT PAU), 08041 Barcelona, Spain; ^2^Psychiatry Department, Santa Creu i Sant Pau Hospital, 08025 Barcelona, Spain; ^3^Center of Biomedical Network Research on Mental Health (CIBERSAM), 28029 Madrid, Spain; ^4^NTT DATA, 08005 Barcelona, Spain; ^5^Mental Health Division, Althaia Foundation, 08241 Manresa, Spain; ^6^TIC Salut Social Foundation, Ministry of Health of Catalonia, 08009 Barcelona, Spain; ^7^Digital Health Unit, Santa Creu i Sant Pau Hospital, 08041 Barcelona, Spain; ^8^Digital Health, Evidenze, 08005 Barcelona, Spain; ^9^Mental Health and Psychiatry Department, Vic Hospital Consortium, 08500 Vic, Spain; ^10^Institute for Research and Innovation in Life and Health Sciences in Central Catalonia (IRIS-CC), 08500 Vic, Spain

**Keywords:** schizophrenia, treatment-resistant, patient journey, patient experience, stakeholder participation, recovery

## Abstract

**Background:**

Schizophrenia is a heterogeneous mental health disorder associated with severe disability. Approximately 30% of patients do not respond to pharmacological treatment, a condition known as treatment-resistant schizophrenia (TRS). Emerging digital solutions could help to improve the treatment of this population. Although the importance of characterising the patient journey (PJ) is widely recognised, and previously published in schizophrenia, this has never been done in patients with TRS to identify their specific needs and select digital approaches to fill the healthcare gaps. Therefore, this study aimed to (1) characterise the PJ in patients with TRS, (2) determine the key needs of these patients, and (3) identify digital solutions that could help to address those needs.

**Methods:**

Three focus groups were constituted: (1) patients with TRS (n = 6); (2) informal caregivers (n = 4); and (3) social/healthcare professionals (n = 16). An advisory board (n = 11) was also created. We used the PJ and patient experience (PEx) methodologies, which place the user experience at the centre of the process. A five-step process was used to define the PJ, to identify patient and caregiver archetypes, to determine the needs and preferences of patients and caregivers, and to identify solutions (technological and others) to address those needs.

**Results:**

We identified the archetypes of patients with TRS and informal caregivers. Nine stages of the PJ were identified: previous symptomatology; emergency care; hospitalization; therapeutic guidelines; outpatient care; diagnosis; disorder control; exacerbations; and risk behaviours. Six key needs were identified: better care during emergencies; improved understanding of the disorder and adverse events; better communication during diagnosis; better control and monitoring of the disorder; better identification of early warning signs; and immediate professional attention. Twenty-six specific initiatives aimed at improving the PEx and care processes were defined.

**Conclusions:**

This study characterised the PJ in patients with TRS. The findings of this study reveal the key areas of the recovery process that need improvement. Importantly, we developed a set of twenty-six specific initiatives to improve clinical outcomes. The main need identified by participants was for non-pharmacological interventions.

**Trial Registration:**

ClinicalTrials.gov NCT05345977.

## Introduction

Schizophrenia is a mental disorder that affects approximately 24 million people worldwide [1]. This disorder is associated with high disability rates and is primarily characterised by distortions from reality such as delusions and hallucinations (positive symptoms), social isolation and apathy (negative symptoms), and cognitive impairment [2]. Despite advances in pharmacological treatment for schizophrenia, up to one-third of individuals do not respond adequately to treatment [3], a condition known as treatment-resistant schizophrenia (TRS) [4]. This condition imposes a financial burden that is 3-to 10-fold higher than that of non-TRS [5], as well as a significant and prolonged clinical and humanistic burden on patients and their caregivers [6]. Caregivers are the most involved in the life of the patients by filling the gaps in healthcare resources, managing relapses and attending to patients’ daily needs [7]. As a result, caregivers often experience significant disruptions in their social and professional lives [8].

In order to optimize results in treatment for TRS, psychosocial interventions such as cognitive behavior therapy have been considered, but the efficacy remains modest [9]. Moreover, these interventions are not widely available and often not adequately integrated into healthcare systems [10]. These limitations need to be addressed, and novel strategies, such as digital health interventions (DHI), have been developed with the aim of achieving better outcomes for patients [11].

According to the systematic review by Firth and Torous [12], DHI can be feasible and acceptable for patients with schizophrenia, and preliminary findings have shown efficacy to improve self-management of schizophrenia. However, in order to develop effective DHI that enhance quality of care, it is essential to identify potential gaps in current clinical practice and the impact of DHI from the perspective of patients and their informal caregivers [13]. This is especially important in patients with complex conditions such as TRS, as these patients have to face complexity and fragmentation in care, and would likely benefit from an integrated care approach [14]. Individuals with TRS face significant challenges, such as complex treatment regimens, side effects from medication, or lack for personalised care. The complexity of TRS is not supported by a structured care pathway that addresses their long-term management needs [15]. The analyses of the patient care pathway for TRS, would help to identify critical touchpoints where care may be inadequate or fragmented.

In recent years, two different but complementary approaches to understanding patient perspectives have gained prominence, the patient journey (PJ) and the patient experience (PEx). The PJ seeks to describe the route that a patient follows within the social and health care system after onset of the first symptoms (or diagnosis) [16]. The PEx is a description of how patients experience their disease and the consequences of their condition on their lives, starting with the initial awareness of symptoms through all stages of the disease [17]. These methodologies allow clinicians to evaluate the entire therapeutic process by placing the experiences of patients and informal caregivers at the centre of the process. This information can help to identify solutions based on the needs and preferences of patients and their informal caregivers. These methodologies allow us to obtain an accurate description of the care pathway, which in turn can lead to better quality care by improving risk-adjusted outcomes, promoting patient safety, increasing patient satisfaction, and optimizing resource usage [18].

Several studies have evaluated the PJ in schizophrenia [18, 19]. Mohr *et al*. [18] applied this methodology to identify the factors that are essential to the effective management of schizophrenia, including early detection and intervention programs, timely intervention, and relapse prevention. In a similar study, Percudani *et al*. [19] identified specific areas related to early detection and long-term management needed to be further implemented.

In this context, the aims of the present study were to obtain the perspectives of patients with TRS and informal caregivers to better understand the PJ and PEx, and to define appropriate technological initiatives that respond to patients’ real needs. This study is part of the European eMotiph research project (clinicaltrials.gov; NCT05345977, https://clinicaltrials.gov/study/NCT05345977), whose aim is to develop an innovative mHealth solution to empower patients with TRS based on their PJ.

## Methods

This was a qualitative study designed to characterise the PJ and PEx through focus groups comprised of patients with TRS, informal caregivers, and social/healthcare professionals. The present study was conducted over the period from May to June 2019.

### Participants

Participants included patients with TRS, informal caregivers, and social and healthcare professionals working in mental health care within the public healthcare system in Catalonia, Spain. An advisory board comprised of healthcare professionals, healthcare planners, and patient associations in the field of mental healthcare was formed to assess the viability of the proposed solutions.

Patients and informal caregivers were recruited from the outpatient service of the Psychiatry Department at the Santa Creu i Sant Pau Hospital (SCSPH) in Barcelona, Spain. Ten patients treated in the Treatment-Resistant Schizophrenia Programme at SCSPH were invited to participate, but only six accepted. Social and healthcare professionals were recruited from the SCSPH and other centres in the Barcelona area.

Eligibility criteria for patients were based on the operational definition of TRS defined by Howes *et al*. [4], as follows:

(1) At least two failed adequate trials with different antipsychotics (chlorpromazine-equivalent doses ≥600 mg/day for ≥6 consecutive weeks) and ≥4 points on the Clinical Global Impression-Severity Schizophrenia (CGI-SCH [20]) and ≤50 on the Global Assessment of Functioning (GAF [21]) scales; or

(2) Patients meeting criteria for TRS and receiving ongoing treatment with clozapine who score ≥4 points on the CGI-SCH and ≤50 on the GAF scales.

Exclusion criteria for patients were to reject participants younger than 18 years old, and with the presence of physical inability to answer the questions.

### Methodolo

The PEx methodology seeks to understand two main areas of the PEx: (1) experience with the disorder (i.e., clinical burden, use of health and social care services; treatment-related side effects; and relapse, among other aspects) and (2) experience with their family, social or work environment. In this regard, the PEx and PJ methodologies, taken together, provide a comprehensive picture of the PExin the following areas: (1) the different stages of the disorder, (2) the points of contact between the patient and the health and/or social care system, (3) the therapeutic interventions(TI), (4) their everyday experience living with the disease (i.e., how they manage the disorder at home) and, (5) the consequences of the disorder on their social relations (i.e., how the disease influences their social or work relations and how they experience these relationships). This methodology helps to identify key gaps and pain points in the PJ by examining the emotional and behavioural aspects of patients’ daily lives, and by assessing their attitudes, beliefs, and perceptions towardTI, thereby revealing opportunities for improvement. The PEx method assesses healthcare interventions from the perspective of patients and informal caregivers to identify beliefs, attitudes, behaviours, emotions, and interactions previously unknown to the healthcare system. In turn, this provides a more comprehensive understanding of the patient to improve the care process.

In this study, we followed a five-step process: (1) preparation; (2) identification of patient and informal caregiver archetypes; (3) definition of the PJ; (4) definition of initiatives; (5) evaluation of initiatives to improve the PJ.

A series of seven workshops (WS) involving social/healthcare professionals, patients, informal caregivers, and the advisory board were held in a meeting room at SCSPH (description in Table 1). The WS were handled by a moderator and a coordinator. The former, responsible for proposing the subjects for discussion and providing direction to the conversation, was a psychologist with over 18 years’ experience in qualitative research methods. The latter, a healthcare strategy consultant with over 10 years of experience, welcomed the participants, gave out questionnaires, recorded the conversations and took notes. In order to gather the information of participants and reduce guiding bias, a standardized interview outline was used (**Supplementary material 1**).

**Table 1.** **List of workshops and aims**.

Workshop	Target	Aims
WS1	Professionals	Definition of archetypes and preliminary PJ (initial mapping of patient and caregiver interactions with the healthcare system)
WS2	Patients	Definition of PJ
WS3	Caregivers	Definition of PJ
WS4	Professionals	Development of a list of proposed initiatives
WS5	Technology experts	Expert insights about the proposed initiatives
WS6	Advisory board	Evaluation of initiatives
WS7	Professionals	Prioritisation of initiatives

Abbreviations: WS, workshops; PJ, patient journey.

The techniques used in each step of the process are described in detail below:

Step 1. Introductory session.

The aim of the introductory session was to develop a global vision of the PJ by gathering relevant information about the disorder and how it is managed by the healthcare system and by patients. This information was then used to design the WS. The project research team conducted semi-structured interviews with patients with TRS and with healthcare professionals from different areas involved in the management of patients with TRS. The interviews were held in the outpatient service of the Psychiatry Department at SCSPH.

Step 2. Identification of patient and informal caregiver archetypes.

In this step, social/healthcare professionals participated in WS1 (Table 1), aimed at identifying the most common profiles (“archetypes”) of the patients and their informal caregivers based on their patterns of behaviour, emotions, routines, preferences, and expectations about the disease and theTI. A survey was administered to obtain the description of the archetypes.

Step 3. Definition of the PJ.

This step involved an in-depth analysis of the life circumstances of the patients and their use of healthcare resources. We considered clinical factors as well as the psychological and social elements shaping their environment. This approach can provide valuable insights to help develop personalized solutions for each patient. To develop a comprehensive picture of the PJ, we organized three WS (WS1–3, Table 1) to obtain a wide range of information and feedback from social/healthcare professionals, patients, and informal caregivers to explore the PJ from every possible angle.

Step 4. Defining the initiatives.

After we completed the first three steps described above, the participating social/healthcare professionals developed a list of initiatives (and their digital/analogue format) aimed at improving the treatment of these patients by addressing their needs. In particular, these initiatives sought to address treatment-related gaps in the current healthcare system (WS4, Table 1). We organized an additional WS (WS5, Table 1) with technology experts experienced in designing and developing DHI for mental health. The objective was to identify best practices and gather expert insights on the proposed digital initiatives for the eMotiph project.

Step 5. Evaluation of the proposed initiatives.

The last step was to evaluate and prioritise the set of 26 initiatives. The advisory board examined the preliminary list of initiatives and assessed the feasibility and suitability of the final list (WS6, Table 1). Then, social/healthcare professionals, patients and informal caregivers were asked to rate the proposed solutions on a 4-point scale (0 to 3 points) to determine the six initiatives that they believed would most improve the quality of healthcare received, and the daily management of the disorder. They were also asked to select one proposal that they considered to be the most important initiative (supervote).

For the participating social/healthcare professionals, the prioritization process was carried out during WS7 (Table 1). For the patients and informal caregivers, prioritization was assessed by means of an online survey.

The average length of the sessions was 60 minutes for the interviews and 180 minutes for the WS.

The sessions were recorded using an audio recorder and subsequently transcribed, enabling a systematic analysis of the emergent information through conventional content analysis. The process of conventional content analysis involves the systematic examination of data, often through repeated readings, with the aim of achieving comprehensive understanding and developing a holistic perception of the entire dataset. Subsequently, the data are processed in order to derive codes; this is achieved by reading each word in turn. The process of identifying the most salient words in a text is initiated with the objective of capturing the key concepts and ideas expressed within the text. Specifically, the steps for the conventional content analysis were as follows: organising and coding; categorisation; abstraction (themes creation); interpretation; and validation. The final step consisted of intercoder reliability checks, in which two researchers coded the same data and compared their results, and iterative analysis, in which researchers returned to the data to refine categories and themes.

## Results 

A total of six patients with TRS (three women, three men) participated in the focus group (Table 2). The mean patient age was 43 years (standard deviation [SD]: ±5.82). The mean duration of the disorder was 18 years (SD: ±8.59). Four informal caregivers (one man, three women) were recruited. A total of 16 social/healthcare professionals (15 women) from the fields of psychiatry, psychology, nursing and social work participated in the WS. The advisory board was comprised of 11 people (six women).

**Table 2.** **Demographic data, duration of disorder and TRS criteria of included patients**.

Participant	Sex	Age	Duration disorder (years)	Failed trial 1	Failed trial 2	Current treatment	CGI-SCH	GAF
Patient 01	M	44	17	aripiprazole 55 mg/d	paliperidone IM 300 mg/4 w	clozapine 250 mg/d	5	40
Patient 02	W	45	18	haloperidol 20 mg/d	olanzapine 30 mg/d	clozapine 300 mg/d	6	30
Patient 03	W	44	18	aripiprazole 45 mg/d	perphenazine 50 mg/d	clozapine 50 mg/d; ziprasidone 180 mg/d	6	40
Patient 04	M	35	18	amilsulpride 1200 mg/d	quetiapine 600 mg/d; ziprasidone 160 mg/d	aripiprazole 20 mg/d; quetiapine 150 mg/d	5	40
Patient 05	W	37	6	olanzapine 30 mg/d	aripiprazole 45 mg/d	clozapine 300 mg/d	6	30
Patient 06	M	51	33	haloperidol 40 mg/d	risperidone IM 100 mg/15 d	clozapine 300 mg/d; clopixol IM/5w	5	40

M, man; W, woman; IM, intramuscular; CGI-SCH, Clinical Global Impression-Severity Schizophrenia; GAF, Global Assessment of Functioning.

### Identification of Archetypes (Patients and Informal Caregiver)

The social/health care professionals identified the most common profile of patients with TRS and informal caregivers (archetypes). Patients were classified into archetypes (Table 3) according to two key variables: (1) level of insight (high or low) and (2) proactive monitoring of the treatment (positive or negative).

**Table 3.** **Patient archetype**.

		Insight	
		+	–
Proactive Monitoring of the Treatment	+	Patients who are active, attend scheduled visits, and show high adherence to the prescribed treatment.	Patients who attend scheduled visits and adhere to treatment but passively. These patients are more prone to stop taking their prescribed medications.
	–	Patients whose behaviour is passive and are more prone to disengaging from treatment.	Patients unwilling to adhere to the prescribed treatment and lack a social network or friends.

Informal caregivers were classified into two archetypes according to two key variables: (1) acceptance of the disorder, and (2) proactivity in care monitoring (Table 4).

**Table 4.** **Informal caregiver archetype**.

		Acceptance	
		+	–
Proactivity in Care Monitoring	+	Caregivers who are active and keep informed of the resources available to patients.	Caregivers who refuse to accept the disorder, but help in monitoring and treatment.
	–	Caregivers who accept the disorder, but do not actively engage in the treatment process.	Caregivers who refuse to accept the disorder, and do not participate in the treatment process. This affects patient compliance.

### Definition of the PJ

Based on the analysis of the data obtained through the WS (patients, informal caregivers and social/healthcare professionals), we identified and mapped nine stages of the disorder, as follows: (1) previous symptomatology; (2) emergency care; (3) hospitalization; (4) therapeutic guidelines; (5) outpatient care; (6) diagnosis; (7) disorder control; (8) exacerbations; (9) risk behaviours. A total of 33 different “experiential moments” with the healthcare route were identified during the PJ, distributed through the nine stages. The **Supplementary Fig. 1** provides a comprehensive overview of the PJ, including the experiences and expectations of patients with TRS and informal caregivers through the nine stages of the journey.

The experiences that patients considered to be the most important where matched with stages 2, 4 and from 6 to 9, as shown in Table 5. These stages contain the six key needs for intervention in TRS.

**Table 5.** **Most important experiences in the patient journey**.

Stage 2		Emergency Care
	Experience 8	“I receive care in the psychiatric emergency unit”
Stage 4		Therapeutic Guidelines
	Experience 13	“I follow the treatment guidelines”
	Experience 14	“I have adverse events”
	Experience 15	“I do not respond to the treatment”
Stage 6		Diagnosis
	Experience 21	“They give me a primary diagnosis”
	Experience 22	“They give me a definitive diagnosis”
Stage 7		Disorder control
	Experience 26	“I have a social life and a work life”
	Experience 27	“I manage my persistent symptoms”
Stage 8		Exacerbations
	Experience 29	“I identify my symptoms”
	Experience 30	“Others detect my symptoms”
Stage 9		Risk Behaviours
	Experience 31	“I self-harm”
	Experience 32	“I attack others”
	Experience 33	“I consume toxic substances”

The co-morbidities experienced by patients with TRS (substance abuse, depression and anxiety) were identified at certain stages of the care continuum, including previous symptomatology, hospitalization, exacerbations and risk behaviours. However, substance abuse was the only issue reported as being key to recovery. Patients recognised that substance use exacerbates their symptomatology, and that they require assistance to overcome these risk situations.

Table 6 summarizes the expectations of the different participants (patients, informal caregivers, social/healthcare professionals) with regards to the interventions.

**Table 6.** **Expectations of patients, informal caregivers, and social/healthcare professionals**.

Patient expectations					
Contact with professionals	Information	Treatment	Prevention	Health guidelines	Follow-up
To ensure the continuity of professionals	To receive personalized care for a better understanding of the disorder	To receive immediate support	To identify symptoms early that may lead to an exacerbation	To keep patients active through activities tailored to their needs	To receive support in adhering to the treatment plan, including follow-up visits
Informal caregiver expectations					
Contact with professionals	Information	Treatment	Prevention	Psychological support	
To know where to access specialized care	To learn how to recognize warning signs	To receive support if the patient discontinues medication	To have sufficient information to distinguish early symptoms from typical adolescence behaviour	To have psychological support for managing daily life as a caregiver	
Social/Healthcare professional expectations					
∙ Telemonitoring of physiological variables in patients’ daily lives					
∙ Interdisciplinary management during hospitalization					
∙ Holistic self-management of the disorder: treatment, social relationships, and physical activity					
∙ Prevention of exacerbations					
∙ Prevention of risk behaviour					
∙ Positive stimulus management					

### Definition and Evaluation of Initiatives for Improvement

Based on the data gathered from the participants during the WS, a total of 26 initiatives aimed at improving care processes and patient experiences were drawn up and categorized into seven thematic areas (Table 7). Then a second list was made with regards to the format of those proposals (digital or analogue).

**Table 7.** **Initiatives and proposal for digital and analogue transformation grouped by theme**.

Information and Public Awareness (IPA)	
Initiatives targeting the general public to reduce social stigma, increase understanding of schizophrenia, and raise awareness about the patient experience and their environment.	
Initiative	Digital/Analogue Transformation
IPA.1- Schizophrenia awareness campaign	IPA.1- Communication strategies delivered through social networks and/or the media. The material may consist of playful interactive materials—or famous people (actors, etc.) who share their experience with a family member who has the disorder
Health Education (HE)	
Training initiatives targeting the following: healthcare professionals, social workers, schools, patients, informal caregivers, and community associations. These initiatives aim to improve the early identification of symptoms, diagnosis, and management of the disorder (patients and informal caregivers).	
Initlative	Digital/Analogue Transformation
HE.1- Health education programme in schizophrenia	HE.1- Online and in-person educational programme on schizophrenia for professionals, patients, caregivers, schools and associations
HE.2- Collaboration with community agents (associations)	HE.2- Online and in-person programme with community associations (healthy lifestyle workshops)
HE.3- Educational programmes on substance use and the association between substance use and mental health	HE3- Online and in-person educational and interventional programme on risk factors for substance use and schizophrenia
HE.4- Provide reliable information on the disorder for patients and informal caregivers	HE.4- Digital platform with complete, well-validated information about the disease, including a chatbot to answer questions and provide recommendations.
HE.5- Interventions to promote well-being among adolescents and young adults	HE.5- Online and in-person educational and interventional programme for adolescents and young adults, to promote a healthy lifestyle and prevent substance use.
HE.6- Diagnosis and treatment communication	HE.6- Improve support in the diagnostic process (space for questions, promote empathy, etc.)
Prediction and Prevention (PP)	
Technological initiatives designed to anticipate and prevent certain aspects of the disorder (onset, relapse, treatment response, etc.) to support decision making and enable a personalized preventive intervention.	
Initiative	Digital/Analogue Format
PP.1- Automatic recognition of symptoms	PP.1- Device with sensors (e.g., bracelet) to detect warning signs (e.g., disturbed sleep patterns)
PP.2- Early detection of symptoms in the primary care and school settings	PP.2- Detection of early signs of schizophrenia (primary care professional)
PP.3- Digital phenotyping	PP.3- Device with sensors (e.g., smartwatch) to adapt treatment to the patients’ unique characteristics
Therapeutic Interventions (TI)	
Initiatives aimed at implementing, complementing and/or improving therapeutic interventions in patients and informal caregivers to improve certain aspects of the disorder (patients) or the challenging effects of caregiving (informal caregivers).	
Initiative	Digital/Analogue Format
TI.1- Strengthen psychological support for the patient	TI.1- Videoconferencing tool to conduct psychological visits (scheduled and unscheduled)
TI.2- Support strategies aimed at informal caregivers	TI.2- Psychological support for informal caregivers
TI.3- Exposure therapy to help patients cope with delusional symptoms and emotional dysregulation	TI.3- Digital platform to improve cognitive deficits
TI.4- Therapeutic alternatives	TI.4- Digital platform to improve social skills
TI.5- Research agenda and patient participation	TI.5- Virtual reality to help patients manage emotions and cope with auditory hallucinations
Comprehensive Illness Management (CIM)	
Initiatives to promote continuous monitoring of care and to empower patients and informal caregivers in managing the disorder in everyday life.	
Initiative	Digital/Analogue Format
CIM.1- Continuous tracking of patient status (monitoring)	CIM.1- Continuous monitoring of the mental health condition through a device that gives patients alerts and recommendations
CIM.2- Personalized recommendations for occupational and cultural activities	CIM.2- Recommendation for cultural, leisure, or occupational activities according to patient preferences
CIM.3- Personalized plans for healthy lifestyles	CIM.3- Personalized plans of activities and materials to promote a healthy lifestyle
CIM.4- Online consultations	CIM.4- Video/telephone consultations or onsite visits with a psychologist or psychiatrist
CIM.5- Adherence to healthcare processes and pharmacological treatment	CIM.5- A device designed to improve treatment adherence (reminders, recommendations, etc.)
Humanization of Care (HC)	
Initiatives related to the definition and optimization of processes and protocols to improve the experience of patients and informal caregivers, especially at key time points.	
Initiative	Digital/Analogue Format
HC.1- Humanization of the care continuum	HC.1- Shared decision-making (patient and psychiatrist)
HC.2- Personalized care in the diagnostic phase	HC.2- Training for professionals on the optimal approach to giving the diagnosis to the patient
HC.3- Humanization of hospitalization	HC.3- Improving the hospital experience to make it more comfortable, (e.g. availability of leisure activities) and respecting patient preferences (diet, clothing, etc.)
HC.4- Interdisciplinary management of hospitalization	HC4- Development of a tool to provide interdisciplinary, well-protocolized, coordinated care during emergency hospitalizations
Peer and Community Support (PCS)	
Initiatives to foster support among peer groups (informal caregivers and patients) with the support of public community resources.	
Initiative	Digital/Analogue Format
PCS.1- “Expert” patient and informal caregiver	PCS.1- Provide selected patients with training to enable them to support to other patients
PCS.2- Support networks for patients and informal caregivers	PCS.2- Support networks for informal caregivers and patients to contact and share experiences

Once the initiatives were identified, they were linked to the nine stages of the disorder identified with the mapping process of the PJ (see **Supplementary Fig. 1**). This made it possible to match the six key needs identified with the corresponding digital solutions (Table 8).

**Table 8.** **The key needs and corresponding digital initiatives theme**.

Key Needs	Digital Initiatives Theme
Better care during emergencies	Humanization of care
	Health education
Improved understanding of the disorder and adverse events	Therapeutic interventions
	Comprehensive illness management
Better communication during diagnosis	Health education
	Therapeutic interventions
Better control and monitoring of the disorder	Health education
	Therapeutic interventions
	Comprehensive illness management
Better identification of early warning signs	Prediction and prevention
	Humanization of care
	Peer and community support
Immediate professional attention	Health education
	Prediction and prevention
	Humanization of care

**Fig. 1.**
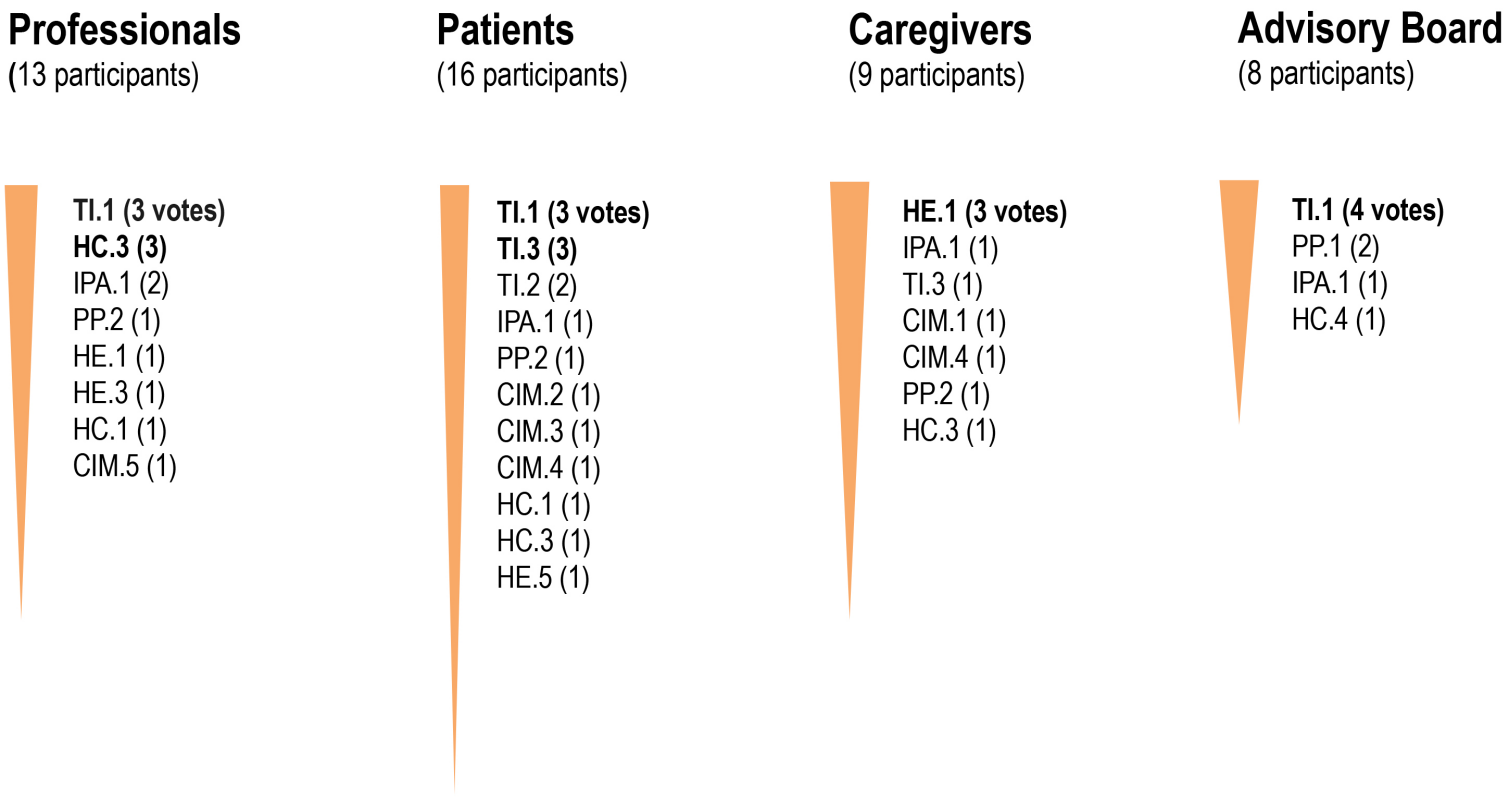
**The digital/analogue initiatives that received the most votes by each group of participants**. CIM.1: Continuous monitoring of mental health condition through a device that gives patients alerts and recommendations; CIM.2: Recommendation for cultural, leisure or occupational activities according to patient preferences; CIM.3: Personalized plans of activities and materials to promote a healthy lifestyle; CIM.4: Video/Telephone consultations with a psychologist or psychiatrist; CIM.5: A device designed to improve treatment adherence; HC.1: Shared decision-making; HC.3: Improving the hospital experience; HC.4: Development of a tool to provide an interdisciplinary, well-protocolized, coordinated assistance during emergency hospitalization; HE.1: Online and in-person educational programme on schizophrenia for professionals, patients, caregivers, schools and associations; HE.3: Online and in-person educational and interventional programme on risk factors for substance abuse and schizophrenia; HE.5: Online and in-person educational and interventional programme for adolescents and young adults to promote a healthy lifestyle and prevent substance abuse; IPA.1: Communication strategies delivered through social networks and/or the media; PP.1: Device with sensors to detect warning signs; PP.2: Detection of early signs of schizophrenia (primary care professionals); TI.1: Videoconferencing tool to conduct psychological visits; TI.2: Psychological support for caregiver; TI.3: Digital platform to improve cognitive deficits.

The results of the prioritization process are shown in Fig. 1.

The initiative that received the most votes by social/healthcare professionals, patients with TRS, and the advisory board was the development of a videoconferencing tool designed to offer online psychological care visits (initiative TI.1), including scheduled and unscheduled visits.

For caregivers, the initiative that received the most votes was an online and in-person educational programme on schizophrenia designed for professionals, patients, caregivers, schools, and associations (initiative HE.1).

## Discussion

Advances in the pharmacological treatment of schizophrenia have greatly improved Quality of life(QoL) in these patients [22]. However, a high proportion (up to 30%) of patients remain resistant to pharmacological treatment [3]. In this patient subset, management of the disorder is highly challenging. In order to effectively improve QoL in patients with TRS, it is crucial to gain a better understanding of the experiences, needs, and preferences through the entire diagnostic and therapeutic process.

In this study, we mapped out the PJ of a small group of patients with TRS. The PJ methodology has many important advantages, most notably its focus on humanizing care, enhancing social esteem, and providing therapeutic support beyond pharmacological treatment. This approach could also be used to develop a new model of care to promote the holistic recovery of the individual. In this context, it is essential to assess patients’ direct experiences to identify complementary therapeutic practices (such as digital solutions) that are fully aligned with the needs of individuals with mental health disorders. The information obtained through this assessment can help to determine whether—and to what extent—digital health tools could improve their treatment. The mapping process revealed key needs in six of the nine stages of the disorder, as follows: emergency care; therapeutic guidelines; diagnosis; disease control; exacerbations; and risk behaviours. Similarly, through the WS, we identified a total of 26 initiatives aimed at improving the PJ. Then we matched the initiatives that were most appropriate to each of the six stages for which key needs were identified.

### Emergency Care 

In this study, patients and informal caregivers both reported positive and negative experiences related to emergency care. In general, the experience with emergency care depended on the level of personal care received from healthcare professionals, ambulance services, and police forces. In the same way, empirical research suggests that emergency care is often experienced as negative, stressful, and even traumatic [23]. This is relevant given that studies show that people with schizophrenia are at high risk of exposure to trauma and developing post-traumatic stress disorder, which can, in turn, reduce adherence to pharmacological treatment [24, 25, 26]. Based on the PJ findings, several initiatives in the area of *Humanization of care (HC) *and* Health education (HE)*, addressed to police officers and healthcare professionals working in ambulances and emergency services were proposed (Table 7).

### Therapeutic Guidelines

Patients and informal caregivers both indicated a need for a better understanding of how to prevent and manage adverse events in the current study. Both groups also noted that it should be possible to interrupt treatment in the presence of adverse events or if the treatment is not having the desired effect. This is relevant given that studies show that medication side effects can decrease adherence [27, 28], and a lack of response to treatment can be a source of frustration and burden for patients and caregivers alike [29]. The two initiatives most closely related to the Therapeutic guidelines needs identified in the study were *TI* and *CIM*.

### Diagnosis

In this study, patients expressed feelings of shame, guilt, and sadness related to their diagnosis. Similarly, in a survey involving patients with schizophrenia and their caregivers, Thomas *et al*. [30] found that the diagnosis can harm patients in many ways through the stigma associated with schizophrenia, which may have a wide-ranging negative impact on patients’ lives such as reducing their ability to find work and support their families. The initiatives related to diagnosis were grouped into the topics *HE* and *TI*.

### Disease Control 

In the current study, the key points identified in the disease control stage were related to recovery (clinical and functional outcomes), a finding that has been described in other studies of patients with schizophrenia [31, 32]. The initiatives related to this stage were in *HE*, *TI*, and *CIM*.

### Exacerbations 

In the current study participants were interested in learning how to identify early warning signs and how to obtain prompt professional support. In this sense, Allan *et al*. [33] carried out a study involving focus groups comprised of mental health staff, caregivers, and patients on the psychosis spectrum. The study found that detection of early warning signs is a key instrument to prevent potential relapses. The initiatives were classified under the topics *Prediction and prevention (PP)*, *HC* and *Peer and community support (PCS)*.

### Risk Behaviours

The need that received the highest support (in terms of votes) was immediate professional care. The initiatives most closely related to this need were included in the topics *HE *(addressed to police and security staff), *PP*, and *HC*.

The integration of DHI in mental health care systems could enhance patient engagement, accessibility and treatment outcomes. With this perspective in mind, the current study identified digital solutions that could help address the needs of patients and caregivers. A total of 26 initiatives were drawn up and categorized in seven thematic areas: Information and public awareness, HE, PP, TI, CIM, HC and PCS. The initiative that received the most votes from patients with TRS, social/healthcare professionals, and the advisory board was the need to strengthen psychological interventions. Despite the consistent body of evidence supporting the efficacy of psychological interventions for psychosis [34], the implementation of these interventions in mental health care remains limited due to the current dominance of the biological model of care, a lack of resources, and a shortage of trained staff among others factors [10].

The identified initiatives may have full potential to enhance scalability and quality of mental health services, but their development implies more than simply designing a service. On one hand, it would necessitate a transformation in the organisational culture of the health sector. For example, involving patients and informal caregivers can help adapt innovative solutions throughout the care process, ensuring that the entire process—from diagnosis to treatment—aligns more closely with the real needs of patients. This approach can lead to a better care experience. However, the integration of shared decision-making into mental healthcare is still in its early stages, and requires further research [35, 36]. Healthcare professionals need to be equipped with the knowledge and confidence to incorporate digital tools into their clinical workflows. Continuous training programs will be key for acquiring new skills to effectively use digital tools, and to understand and communicate digital outcomes. Besides, healthcare professionals may require guidance on balancing in-person and digital care, ensuring that both approaches complement each other rather than the substitution of traditional approaches. The introduction of new technologies that align with clinical protocols can create effective therapeutic environments, promote resource sustainability, and introduce innovative digital interventions (such as smartphone applications) to strengthen the therapeutic approach. On the other hand, policymakers should ensure that privacy and security issues are met by DHI by establishing clear regulatory frameworks. Additionally, they should incentivize the adoption of DHI through funding policies, and provide the necessary infrastructure and support to guarantee successful implementation.

Based on the findings of the present study, the research team responsible for the eMotiph project will begin the development of a novel digital solution to address the needs of patients with TRS, their informal caregivers and the professionals in the healthcare network, thus facilitating its acceptability and usability. This solution will address the key needs identified in the present study: immediacy and continuity of care; monitoring of physiological variables in patients’ daily life; provision of HE and training programmes to recognize symptoms, early warning signs and risk behaviours; psychological strategies to manage clinical symptoms; and psychological support for informal caregivers. With these premises in mind, and in order to ensure users acceptance, the following list enumerates the primary technological functionalities to be implemented into the eMotiph digital solution: monitoring of physiological variables (e.g., heart rate, steps) and symptoms (e.g., speech pattern, mood); therapeutic modules for healthy lifestyle, treatment adherence and psychological therapy; chatbot with psychoeducational information; questionnaires to assess risk behaviour; promotion of social interaction (e.g., forum); and artificial intelligence software (e.g., personalized care plan, pattern recognition, risk prediction, gamification approach through personalized goals and rewards). In addition, the possibility of integrating the solution into patients’ electronic health records will be studied to facilitate its future implementation in healthcare systems.

### Strengths and Limitations

Due to the limited sample size and highly specific patient population (urban setting; medium-high socioeconomic level), the results of this study cannot be generalized to other settings. These findings should be replicated in a larger study, with a broader social and demographic representation. Another limitation is the application of a cross-sectional design, which may result in missing of temporal variability and be affected by recall bias. Finally, the presence of a potential bias of self-reported data could introduce distortion based on present conditions, misrepresent the timing of past events, or generate social desirability bias. The main strengths of this study include the many novel initiatives aimed at ensuring alignment among all stakeholders towards the same goals. These proposals may help to ensure that the care process is focused on the needs of the patients (and caregivers), particularly the development of innovative digital strategies for the highest priority needs. The implementation of these initiatives would likely improve patient experiences and health outcomes, and make the system more sustainable.

## Conclusions

To our knowledge, this is the first study to describe the PJ in individuals with TRS and their informal caregivers.

This study reveals the most important needs that should be targeted to promote better self-management of the disorder and to improve the user experience, and their transformation in digital initiatives. Improving the organizational culture of the mental healthcare system, providing ongoing training in professionals, and engaging policy makers in the development of regulation for DHI implementation will enhance the integration of mental DHI into clinical practice. Our findings also underscore the importance of integrating non-pharmacological approaches (e.g., psychological support, HE programs) as part of the therapeutic process to promote recovery.

Despite the preliminary nature of these findings, we believe these results can help lay the groundwork for the development of personalized treatments tailored to the needs and expectations of all stakeholders.

## Data Availability

The data that support the findings of this study are available from the corresponding author, upon reasonable request.

## Abbreviations

CGI-SCH, Clinical Global Impression-Severity Schizophrenia; CIM, Comprehensive Illness Management; DHI, Digital Health Interventions; GAF, Global Assessment of Functioning Scale; HC, Humanization of Care; HE, Health Education; SCSPH, Santa Creu i Sant Pau Hospital; IPA, Information and Public Awareness; PCS, Peer and Community Support; PEx, Patient Experience; PJ, Patient Journey; PP, Prediction and Prevention; QoL, Quality of life; TI, Therapeutic Interventions; TRS, Treatment-resistant schizophrenia; WS, Workshops.

## Supplementary Material

Supplementary material associated with this article can be found, in the online version, at https://doi.org/10.62641/aep.v53i6.1959.
