# Supplementary material for: Defining the Patient Journey and Identifying Digital Health Solutions in Treatment-Resistant Schizophrenia
Source: Actas Esp Psiquiatr. 2025 Dec 17;53(6):1208–22. doi: 10.62641/aep.v53i6.1959 (PMC12728537; doi:10.62641/aep.v53i6.1959)
Supplement: Supplementary file 1 [file ActEsp-53-6-1208-1222-s1.zip › Supplementary Fig. 1.pdf]

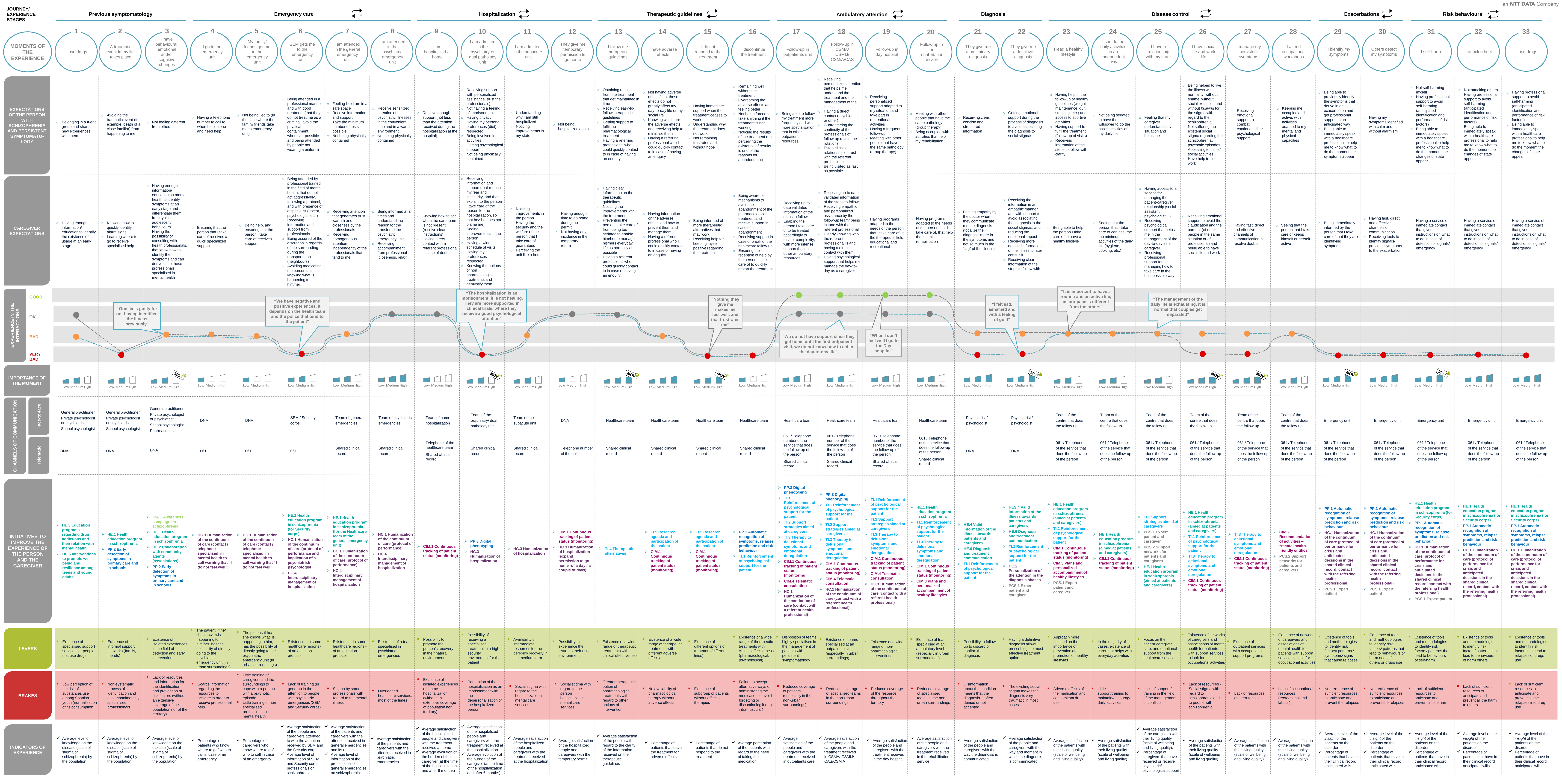

* This Journey has been designed in accordance with the experience collected of the patients, carers and professionals of the healthcare area of the Hospital de la Santa Creu i Sant Pau (Barcelona), thus differences in the contents with regard to other areas of healthcare attention may be found

Key: SEM: System of Medical Emergencies ● CSMA: Centre of Mental Health of adults ● CSMIJ: Centre of Mental Health of Children and Young people ● CSMIA: Centre of Mental Health and Addictions ● CAS: Centre of Attention and Follow-up of drug addictions ● 061: Telephone number for medical emergencies ● DNA: Does Not Apply

Recurring episodes

MOV Moment of truth

Experience of the person with treatment-resistant schizophrenia

Experience of caregiver

CATHEGORIES OF INITIATIVES: IPA - INFORMATION AND PUBLIC AWARENESS; HE- HEALTH EDUCATION; PP - PREDICTION AND PREVENTION; TI - THERAPEUTIC INTERVENTIONS; CIM- COMPREHENSIVE ILLNESS MANAGEMENT; HC - HUMANIZATION OF CARE; PCS- PEER AND COMMUNITY SUPPORT

Fig. 1 Journey and Experience of the People with Treatment-resistant Schizophrenia with the Disease and the Healthcare System - e-Motiph Project*
