# Supplementary material for: Defining the Patient Journey and Identifying Digital Health Solutions in Treatment-Resistant Schizophrenia
Source: Actas Esp Psiquiatr. 2025 Dec 17;53(6):1208–22. doi: 10.62641/aep.v53i6.1959 (PMC12728537; doi:10.62641/aep.v53i6.1959)
Supplement: Supplementary file 1 [file ActEsp-53-6-1208-1222-s1.zip › Supplementary material 1.docx]

**Supplementary material 1- Interview Guide**

Research topics included in the workshops with participants:

- Patients/caregivers’s beliefs related to the disease:
  - experiences throughout the disease
  - expectations and concerns
- Pattern of patient/caregiver interaction with the healthcare system:
  - Definition of stages, moments and interactions with the healthcare system during the evolution of the disease
  - Experiences, perceptions and emotions in relation to the healthcare system and its professionals
  - Levers and brakes
- Patients/caregivers archetypes: Definition of:
  - Patient/caregiver description
  - Attitude towards de disease
  - Needs/expectations
  - Frustrations
- Role of technology as an enabler of improvements in the clinical follow-up received by patients:
  - Identification of moments throughout the process of the disease where technological initiatives could be helpful
  - Suggestions of technological initiatives
